# Urinary cast is a useful predictor of acute kidney injury in acute heart failure

**DOI:** 10.1038/s41598-019-39470-1

**Published:** 2019-03-13

**Authors:** Satoshi Higuchi, Yusuke Kabeya, Kenichi Matsushita, Satoko Yamasaki, Hiroaki Ohnishi, Hideaki Yoshino

**Affiliations:** 1 Division of Cardiology, Department of Internal Medicine II, Kyorin University School of Medicine, Tokyo, Japan; 2 Division of General Internal Medicine, Department of Internal Medicine, Tokai University, Kanagawa, Japan; 3 Department of Home Care Medicine, Saiyu Clinic, Saitama, Japan; 4 Department of Laboratory Medicine, Kyorin University School of Medicine, Tokyo, Japan

## Abstract

Acute kidney injury (AKI) is associated with poor prognosis among patients with acute heart failure (AHF). Early documentation of impaired kidney function through simple examination may provide risk reduction in such patients. The present study aims to reveal an association between cellular casts and hospital-acquired AKI in AHF. This study included patients with AHF who underwent urinalysis, including urinary sediment analysis within 24 hours post admission. AKI was defined as an increase of ≥0.3 mg/dL within 48 hours or ≥1.5 times in serum creatinine level in contrast to baseline creatinine level. In this study, 114 patients with AHF (age, 75 ± 14 years; male, 59.7%) were included. Of them, 40 (35%) developed hospital-acquired AKI. Cellular casts were detected in 30 patients (26%) prior to AKI development and related to hospital-acquired AKI in the multivariate logistic regression analysis (odds ratio, 2.80; 95% confidence interval, 1.04–7.49; *P* = 0.041). In conclusion, cellular casts are observed occasionally in patients with AHF and potentially useful markers for development of AKI during hospitalization.

## Introduction

The growth of elderly population in Japan and improved outcome of patients with cardiac disease due to modern therapeutic progress have led to an increasing incidence of acute heart failure (AHF). Despite improvements in therapy for AHF, its mortality rate remains unacceptable^1,2^. There is a close interrelationship between heart and kidney diseases, known as cardiorenal syndrome (CRS). Impaired kidney function including acute kidney injury (AKI) is common among such patients^3,4^ and is reported to be associated with short– and long–term prognosis in patients with AHF^5,6^. Early documentation of impaired kidney function may provide risk reduction in such patients. However, creatinine is an unreliable marker for detection of early phase of renal impairment in AHF^7^. Furthermore, plasma neutrophil gelatinase–associated lipocalin (NGAL) failed to indicate its usefulness^8^. Until now, various biomarkers for detection of renal impairment were proposed (e.g., kidney injury molecular–1 [KIM–1], cystatin C, interleukin–6, interleukin–8, interleukin–18, N–acetyl–β–D–glucosaminidase [NAG], and liver fatty acid–binding protein [LFABP]), but their usefulness has not been confirmed^9^. Although a combination of such biomarkers may be useful, high cost and complexity are nonnegligible problems. Therefore, early detection of AKI through a simple examination is necessary.

Microscopic examination of the urine sediment is commonly performed, and its clinical value as a noninvasive detector of renal damage has been confirmed^10,11^. For instance, cellular casts can predict subsequent renal relapse in patients with systemic lupus erythematosus^12^. Bagshaw *et al*. reported the potential usefulness of urine sediment examination for the prediction of AKI in sepsis^13^. However, the clinical impact of urinary cast on renal function has not been sufficiently explored in patients with AHF. The present study aimed to clarify the clinical usefulness of urinary casts and whether they detect an early phase of AKI in AHF.

## Results

### Impact of cellular casts in the short term

**Figure 1:**
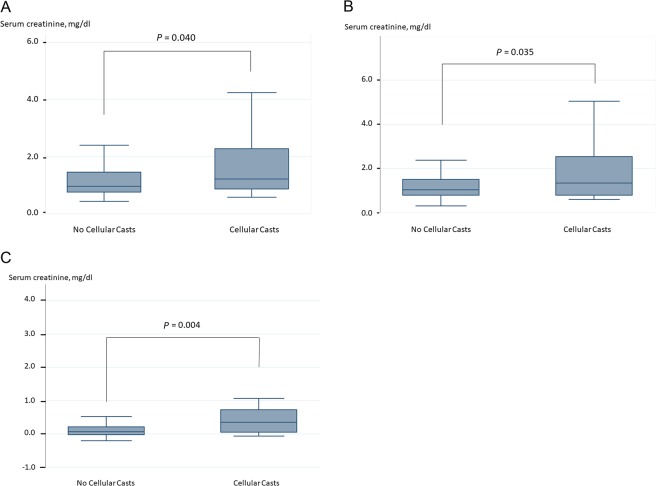
Serum creatinine level between patients with and without cellular casts. (**A**) Serum creatinine level on admission was significantly higher in those who presented with cellular casts. (**B**) Serum creatinine level at discharge was significantly higher in those who presented with cellular casts similar to that on admission. (**C**) Patients with cellular casts indicated an increase in serum creatinine level at the time of discharge, whereas there were few changes in those who presented without them.

**Figure 2:**
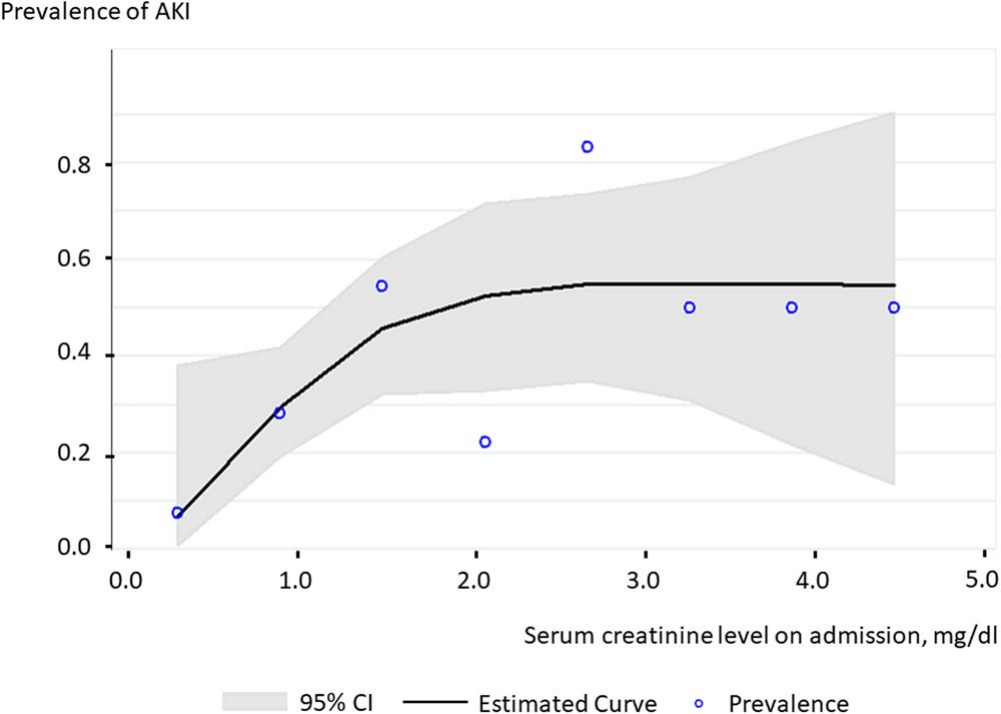
Relationship between the prevalence of hospital-acquired AKI and serum creatinine level. Caption: A cubic spline curve demonstrated that the prevalence of hospital-acquired AKI did not depend on serum creatinine level on admission among patients with serum creatinine level of ≥2.0 mg/dL. AKI: acute kidney injury; CI: confidence interval.

The present study included 114 patients with AHF (age, 75 ± 14 years; male, 59.7%). The patient characteristics are shown in Table 1. Of all patients, 40 (35%) developed hospital-acquired AKI, which developed 5 days (3–10) after admission. Cellular casts were detected in 30 patients (26%). Hyaline cast with and without other casts were found in 30 (26%) and 23 (20%) patients, respectively. Cellular casts were related to a higher serum creatinine level on admission and at discharge, as well as their absolute difference during hospitalization (Fig. 1A–C). Uni– and multivariate logistic regression analyses are presented in Table 2. Cellular casts were significantly associated with hospital-acquired AKI (odds ratio [OR], 2.86; 95% confidence interval [CI], 1.21–6.75; *P* = 0.017), whereas hyaline cast was not (OR, 1.96; 95% CI, 0.77–4.96; *P* = 0.156). The result regarding hyaline cast did not depend on its number. These results persisted after adjustment for age, estimated glomerular filtration rate (eGFR), hemoglobin, and diabetes mellitus (DM) on admission (OR in cellular cast, 2.80; 95% CI, 1.04–7.49; *P* = 0.041; OR in hyaline cast, 1.39; 95% CI, 0.48–4.00; *P* = 0.543). The prevalence of hospital-acquired AKI did not depend on serum creatinine level on admission among patients with serum creatinine level of ≥2.0 mg/dL (Fig. 2).

**Table 1:** Patient Characteristics.

	All (n = 114)	AKI (n = 40)	No AKI (n = 74)	*P* Value
**Patient background**				
Age, years	75 ± 14	78 ± 12	73 ± 15	0.023
Male, n (%)	68 (60)	29 (73)	39 (53)	0.047
Ischemic heart disease, n (%)	42 (37)	21 (53)	21 (28)	0.011
Hypertension, n (%)	71 (62)	27 (68)	44 (59)	0.398
Dyslipidemia, n (%)	33 (29)	9 (23)	24 (32)	0.264
Diabetes mellitus, n (%)	47 (41)	21 (53)	26 (35)	0.072
Hyperuricemia, n (%)	58 (52)	20 (50)	38 (54)	0.721
Left ventricular ejection fraction, %	42 ± 16	44 ± 14	41 ± 16	0.125
NYHA classification				0.099
NYHA II, n (%)	23 (20)	11 (28)	12 (16)	
NYHA III or IV, n (%)	91 (80)	29 (72)	62 (84)	
**Urinalysis**				
Albumin adjusted by creatinine, mg/g·Cre	64 (25–428)	145 (64–565)	43 (24–279)	0.017
β2 microglobulin, μg/L	172 (50–1514)	725 (65–4677)	132 (48–431)	0.015
N-acetyl-β-D-glucosaminidase, IU/L	7 (4–13)	9 (5–15)	5 (3–12)	0.012
Hyaline cast, n (%)	53 (46)	27 (68)	26 (35)	0.001
Epithelial cast, n (%)	20 (18)	12 (30)	8 (11)	0.018
Granular cast, n (%)	25 (22)	13 (33)	12 (16)	0.058
Waxy cast, n (%)	12 (11)	10 (25)	2 (3)	<0.001
Red blood cell cast, n (%)	3 (3)	2 (5)	1 (1)	0.281
Fatty cast, n (%)	7 (6)	7 (18)	0 (0)	<0.001
All casts, n (%)	53 (46)	26 (68)	27 (36)	0.001
Cellular casts, n (%)	30 (26)	16 (40)	14 (19)	0.025
Number of cast types, n	0 (0–2)	1 (0–4)	0 (0–1)	<0.001
Number of cast types ≥ 4, n (%)	12 (11)	11 (28)	1 (1)	<0.001
**Blood examination on admission**				
Creatinine, mg/dl	1.0 (0.8–1.6)	1.3 (1.0–2.1)	0.9 (0.7–1.3)	<0.001
Estimated glomerular filtration rate, ml/min/1.73 m^2^	53 (30–62)	36 (23–50)	56 (36–72)	<0.001
Uric acid, mg/dl	6.9 ± 2.1	7.1 ± 2.4	6.8 ± 2.2	0.787
Hemoglobin, g/dl	11.9 ± 2.1	10.9 ± 2.0	12.4 ± 2.0	<0.001
B type natriuretic peptide, pg/ml	850 (498–1571)	931 (555–1699)	798 (461–1546)	0.454
**Blood examination at discharge**				
Creatinine, mg/dl	1.1 (0.8–1.6)	1.7 (1.2–2.8)	1.0 (0.7–1.3)	<0.001
Estimated glomerular filtration rate, ml/min/1.73 m^2^	52 (34–76)	47 (30–72)	57 (37–77)	0.238
**Medication on admission**				
RAS inhibitor, n (%)	55 (49)	16 (41)	39 (53)	0.238
Calcium channel blocker, n (%)	41 (36)	19 (49)	22 (30)	0.046
β blocker, n (%)	57 (50)	19 (49)	38 (51)	0.790
MRA, n (%)	16 (14)	6 (15)	10 (14)	0.786
Furosemide, n (%)	96 (85)	34 (87)	62 (84)	0.631
Tolvaptan, n (%)	18 (16)	6 (15)	12 (16)	1.000
Antiplatelet therapy, n (%)	56 (50)	24 (62)	32 (43)	0.077
Anticoagulation, n (%)	46 (41)	17 (44)	29 (39)	0.651
**Invasive procedures**				
CAG, n (%)	41 (36)	14 (35)	27 (36)	0.875
Contrast medium of CAG, ml	55 (30–103)	30 (20–60)	72 (40–110)	0.032
PCI, n (%)	10 (9)	5 (13)	5 (7)	0.317
Contrast medium of PCI, ml	70 (50–130)	70 (40–140)	70 (70–110)	0.912
PMI or CRT, n (%)	3 (3)	0 (0)	3 (4)	0.551
Thoracic surgery, n (%)	1 (1)	0 (0)	1 (1)	1.000
**Medication at discharge**				
RAS inhibitor, n (%)	61 (55)	21 (58)	40 (54)	0.672
Calcium channel blocker, n (%)	49 (45)	21 (58)	28 (38)	0.042
β blocker, n (%)	77 (70)	24 (67)	53 (72)	0.595
MRA, n (%)	31 (28)	7 (19)	24 (32)	0.181
Furosemide, n (%)	65 (59)	20 (56)	45 (61)	0.599
Tolvaptan, n (%)	23 (21)	6 (17)	17 (23)	0.618
Antiplatelet therapy, n (%)	51 (46)	21 (58)	30 (41)	0.103
Anticoagulation, n (%)	57 (52)	20 (56)	37 (50)	0.685

AKI: acute kidney injury; CAG: coronary angiography; CRT: cardiac resynchronization therapy; MRA: mineralocorticoid receptor antagonist; NYHA: New York Heart Association; PCI: percutaneous coronary intervention; PMI: pacemaker implantation; RAS: renin–angiotensin system.

**Table 2:** Logistic regression analysis for hospital-acquired AKI.

	Univariate			Multivariate		
	OR	95% CI	*P* Value	OR	95% CI	*P* Value
Age (an increase of 1 year)	1.03	1.00–1.07	0.048	NA		
Male	2.37	1.03–5.43	0.042	NA		
Hypertension	1.42	0.63–3.18	0.399	NA		
Dyslipidemia	0.60	0.25–1.47	0.267	NA		
Diabetes Mellitus	2.04	0.93–4.46	0.074	NA		
Hyaline Cast	1.96	0.77–4.96	0.156	1.39	0.48–4.00	0.543
Cellular Casts	2.86	1.21–6.75	0.017	2.80	1.04–7.49	0.041
All Casts	1.65	1.25–2.18	<0.001	3.08	1.22–7.74	0.017
ß2 microglobulin (an increase of 1 μg/L)	1.00	1.00–1.00	0.131	NA		
N-acetyl-β-D-glucosaminidase (an increase of 1 IU/L)	1.05	1.00–1.10	0.076	NA		
Left ventricular ejection fraction (a 1% absolute increase)	1.02	0.99–1.04	0.240	NA		
NYHA III or IV	0.51	0.20–1.29	0.156	NA		
**Blood examination on admission**						
Creatinine (an increase of 1.0 mg/dl)	1.90	1.19–3.04	0.007	NA		
eGFR (an increase of 10 ml/min/1.73 m^2^)	0.67	0.54–0.82	<0.001	NA		
Hemoglobin (an increase of 1.0 g/dl)	0.70	0.56–0.86	0.001	NA		
B type natriuretic peptide (an increase of 1 pg/ml)	1.00	1.00–1.00	0.333	NA		
**Medication on admission**						
RAS inhibitor	0.62	0.28–1.37	0.239	NA		
Calcium channel blocker	2.25	1.01–5.01	0.048	NA		
ß blocker	0.90	0.41–1.96	0.790	NA		
MRA	1.16	0.39–3.48	0.786	NA		
Furosemide	1.32	0.43–4.05	0.632	NA		
Tolvaptan	0.94	0.32–2.73	0.909	NA		
Antiplatelet therapy	2.10	0.95–4.64	0.066	NA		
Anticoagulation	1.20	0.55–2.63	0.651	NA		

AKI: acute kidney injury; CI: confidence interval; eGFR: estimated glomerular filtration ratio; MRA: mineralocorticoid receptor antagonist; NA: not applicable; NYHA: New York Heart Association; OR: odds ratio; RAS: renin–angiotensin system

Multivariate model was adjusted for age, estimated glomerular filtration rate (eGFR), hemoglobin, and diabetes mellitus.

### Sensitivity and specificity

At cutoff of 4 kinds of urinary casts, specificity was 98.65%, sensitivity was 27.50%, and likelihood ratio was 20.35 for prediction of hospital-acquired AKI. Specificity was 81.08%, sensitivity was 40.00%, and likelihood ratio was 2.11 if cutoff was set as presence or absence of urinary casts.

### Invasive procedure

In the present cohort, 45 patients (39%) underwent any invasive procedure for diagnosis or treatment of underlying cardiovascular diseases; coronary angiography (CAG) alone was performed for 31 patients, elective percutaneous coronary intervention (PCI) on the day different from the date of CAG for 4 patients, ad hoc PCI for 6 patients, pacemaker implantation for 2 patients, cardiac resynchronization therapy for 1 patient, and thoracic surgery for 1 patient. In the procedures of CAG and PCI, the amount of contrast medium was 40 (25–100) ml in CAG alone, 59 (86–102) ml for CAG and 70 (60–100) ml for PCI in elective PCI, and 90 (40–140) in ad hoc PCI. The AKI was detected in 14 of 41 (34%) patients who received CAG or PCI. Although univariate logistic regression analysis demonstrated that no significant association between the amount of contrast medium used in such procedures and AKI, a patient with granular casts, in whom eGFR was 50 ml/min/1.73 m^2^ and injected contrast medium for CAG was 45 ml, developed contrast-associated AKI. The remaining four patients, who underwent invasive procedures such as thoracic surgery, pacemaker implantation, or cardiac resynchronization therapy, did not develop AKI.

### Long–term impact of cellular casts

Of 114 patients, 4 died during hospitalization, and 110 were followed up in the outpatient clinic. The median follow–up duration was 453 days (115–770). Thirty–five patients (32%) had worsening renal function (WRF) at least one time within a year after discharge. The results of the univariate Cox regression analysis are shown in Table 3. Cellular casts were not associated with 1–year WRF (hazard ratio (HR), 1.13; 95% CI, 0.53–2.37; *P* = 0.756).

**Table 3:** Cox regression analysis for 1–year WRF.

	Univariate		
	HR	95% CI	*P* Value
Age (an increase of 1 year)	1.01	0.98–1.03	0.652
Male	0.75	0.38–1.49	0.409
Hypertension	1.22	0.61–2.42	0.578
Dyslipidemia	0.52	0.20–1.34	0.175
Diabetes Mellitus	1.27	0.64–2.51	0.493
Cellular Casts	1.13	0.53–2.37	0.756
All Casts	1.14	0.57–2.26	0.710
Hyaline cast	1.05	0.45–2.42	0.909
ß2 microglobulin (an increase of 1 μg/L)	1.00	1.00–1.00	0.747
N–acetyl–β–D–glucosaminidase (an increase of 1 IU/L)	1.01	0.97–1.05	0.609
Left ventricular ejection fraction (a 1% absolute increase)	1.01	0.98–1.03	0.620
**Blood examination at discharge**			
Creatinine (an increase of 1.0 mg/dl)	1.28	0.95–1.74	0.108
eGFR (an increase of 10 ml/min/1.73 m^2^)	1.00	0.99–1.01	0.612
**Medication at discharge**			
RAS inhibitor	0.86	0.43–1.71	0.666
Calcium channel blocker	1.40	0.72–2.73	0.316
ß blocker	0.63	0.29–1.34	0.230
MRA	0.26	0.09–0.75	0.013
Furosemide	1.54	0.73–3.24	0.256
Tolvaptan	2.21	1.07–4.58	0.033
Antiplatelet therapy	1.23	0.63–2.42	0.548
Anticoagulation	1.13	0.57–2.22	0.728

CI: confidence interval; eGFR: estimated glomerular filtration ratio; HR: hazard ratio; MRA: mineralocorticoid receptor antagonist; RAS: renin–angiotensin system; WRF: worsening renal function.

## Discussion

The present study demonstrated potential usefulness of urinary casts for the evaluation of renal function in AHF. First, they might be an early predictor of AKI during hospitalization. Whereas NGAL was reported to increase one day prior to the increase in serum creatinine level^14^, cellular casts were observed 5 days (median) prior to AKI. The characteristics may be useful for risk stratification and early intervention. Second, urinalysis demonstrated high specificity despite it is a daily, easy, and low–cost examination that differed from other time–consuming and expensive biomarkers.

In the present study, approximately a quarter of all patients presented with any cellular casts. Impaired kidney function includes various lesions such as a tubular, glomerular and vascular, and interstitial. In general, most of cellular casts often appear in patients with acute tubular necrosis (ATN)^11^ rather than those who present with interstitial lesions. Some cellular casts, for instance, red blood cell casts, appear in glomerular disease^15^. Our results suggested that some patients with AHF may present with renal parenchymal lesions, including tubular, glomerular and vascular, potentially or apparently. Renal injury lesions have been seldom mentioned in the topic on AKI in those with AHF. Our study showed that urinary casts would be useful to predict AKI development with high specificity, whereas they might not contribute to rule-out AKI because of its low sensitivity. This weak point is reasonable because there are various etiologies of AKI such as interstitial damage which is not associated with urinary casts.

The pathophysiology of renal insufficiency in patients with AHF has not been sufficiently understood^16^ because the causal relationship in CRS would be different in each case. Appearance of urinary cellular casts might be the result of CRS and reflect renal parenchymal damage due to various mechanisms. A previous study which evaluated urinary NGAL as a marker for tubular damage indicated that patients with heart failure would suffer from a combination of reduced GFR and tubular damage^17^. One of possible mechanisms may be the excessive activation of renin–angiotensin–aldosterone–system (RAAS) which can impair the kidneys via renal vasoconstriction yielding impairment of tubular epithelial cells^18–20^. Furthermore, renal hypoperfusion caused by cardiac dysfunction, venous congestion^21,22^, activation of sympathetic nerve system, and oxidative injury^23^ may also contribute to the renal parenchymal lesions. Renal tubular injury can substantially decrease glomerular filtration by a tubulo–glomerular feedback mechanism^24,25^; the influence might persist for a few weeks, but not for a long–term period as urinary cellular casts were not related to 1–year WRF in the present study. Considering such results, an appearance of urinary cellular casts or hospital-acquired AKI in some AHF patients may be due to reversible causes such as temporal venous congestion and activation of RAAS.

As compared with cellular casts, hyaline cast demonstrated nonsignificant and weaker correlation with hospital-acquired AKI. The result might be due to beta–error because of the small number of patients. Although hyaline cast is generally regarded as nonspecific^26^, the clinical impact may differ in various comorbidities. The clinical implication of hyaline cast in AHF might not be determined in the present study.

The present study included some limitations. First, due to the small number of patients, it is difficult to assess the clinical impact of each cellular cast on hospital-acquired AKI and long-term WRF. Second, patients without urinalysis on admission were not included, which might have yielded selection bias. Finally, urinalysis was performed by a single technician, and the photographs of casts were not routinely taken in our laboratory, which means future verification is difficult using the present data. However, the findings of the present study provided an important clue in the evaluation and treatment of AKI in AHF. Future studies with sufficient number of patients are needed to confirm the results of the present study.

## Conclusion

Cellular casts are observed occasionally in patients with AHF and are potentially useful markers for early documentation of hospital-acquired AKI.

## Methods

### Study population

This study included patients with AHF who were hospitalized in Kyorin University Hospital from January 2015 to April 2018. A diagnosis of AHF is defined as rapid–onset heart failure, new or worsening signs and symptoms of heart failure requiring urgent therapy and hospitalization, based on the Framingham criteria^27^.

### Inclusion criteria

The patients underwent urinalysis including urinary sediment analysis and urine biochemistry within 24 hours post admission for AHF treatment.

### Exclusion criteria

The patients who developed acute coronary syndrome, hemodialysis, or peritoneal dialysis were excluded from the study.

### Data collection

The patients’ background and outcomes were collected through an interview and medical record review. Blood examination, including creatinine, hemoglobin, uric acid, and B type natriuretic peptide was performed on admission in all patients. Serum creatinine was measured by technicians without the knowledge of each clinical course and findings of urinalysis. Follow–up data were acquired by a medical record review or interview at the outpatient clinic.

### Urinalysis

Urinalysis was conducted by a highly experienced clinical technologist without the knowledge of each patient’s clinical course and serum creatinine level within 24 hours after admission. Urinalysis consists of urinary sediment analysis and urine biochemistry, including β2 microglobulin and NAG. In preparing the urine sediment examination, 10 mL of urine was centrifuged at 500 gravity (1500 rpm; radius, 20 cm) for 5 min. Urine sediment was assessed in undyed bright–field observation. Urinary casts were counted in 20–30 fields with high power field (400×). Urinary casts were judged as present if they were observed in at least 1 field. Urinary casts were classified as hyaline, epithelial, granular, waxy, red blood cell, and fatty casts. Any casts except for hyaline cast are described as “cellular casts” in the present study. “All casts” mean both cellular and hyaline casts.

### Definition of technical terms

Hospital-acquired AKI was defined as an increase in serum creatinine level of ≥0.3 mg/dL within 48 hours or ≥1.5 times from the baseline value^28^. One-year WRF was defined as an increase of 0.3 mg/dL or more in the serum creatinine level compared to baseline creatinine level at discharge within a year after discharge^29–31^. Ischemic heart disease was diagnosed if patients presented with any of the following: left anterior descending artery, left circumflex artery, or right coronary artery stenosis ≥75% or left main trunk stenosis ≥50% if they underwent coronary angiography or computed tomography. Dyslipidemia was diagnosed if one of the following criteria was fulfilled: low–density lipoprotein cholesterol level (calculated as total cholesterol – high–density lipoprotein (HDL) – triglyceride/5) of ≥140 mg/dL, HDL cholesterol level of <40 mg/dL, or triglyceride level of ≥150 mg/dL. Hypertension was defined as systolic blood pressure (SBP) of ≥140 mmHg and diastolic blood pressure of ≥90 mmHg^32^. Diagnosis of DM was defined with the following criteria: self–reported diabetes or use of antidiabetic medications based on the questionnaire, fasting plasma glucose level ≥126 mg/dL, or hemoglobin A1c (HbA1c) level ≥6.5%^33^. Hyperuricemia was defined as serum uric acid value of ≥7.0 mg/dL.

### Endpoints

The primary endpoint was hospital-acquired AKI. The secondary endpoint was 1–year WRF.

### Ethical Principles

The study protocol conforms to the ethical guidelines of the 1975 Declaration of Helsinki^34^ in line with the Ethical Guidelines for Epidemiological Research by the Japanese government. The study was approved by the ethics committee at Kyorin University. According to the guidelines, the study satisfied the conditions to waive the requirement for informed consent from individual participants. Therefore, informed consent was waived, which was approved by the ethics committee.

### Statistical analysis

No preceding study evaluated a clinical impact of urinary casts on AKI in patients with AHF. We assumed the number of patients required for the analysis based on the previous study evaluating the clinical value of urinalysis in sepsis although the underlying condition was different. According to the study, the incidence of AKI was 33.3% and 7.4% in patients with and without cellular casts^13^. We assumed the prevalence of cellular casts in reference to the first 40 patients in the present study, which indicated a 30% prevalence rate. Based on 80% power and a significance level of 0.05, 93 patients were required. Numerical data are presented as mean ± standard deviation if the data followed a normal distribution. Otherwise, data are displayed as median and interquartile range (Q1–Q3) values. Categorical variables are expressed as absolute numbers or percentages. Continuous variables were analyzed using the unpaired Student *t*–test or Mann–Whitney U test, while categorical variables were analyzed using Fisher exact test or χ^2^ tests, as appropriate. The association between urinary casts and hospital-acquired AKI was assessed using uni– and multivariate logistic regression analyses and expressed as OR, 95% CI, and *P* value. Variables with a *P* value < 0.10 were retained for the multivariate logistic regression analysis with least absolute shrinkage and selection operator. Sensitivity, specificity, and likelihood ratio of urinary casts for hospital-acquired AKI were calculated. In sensitivity analysis, different assessment was performed. Presence of cellular casts and total numbers of each cast were evaluated. Follow–up began on the date of discharge. The data in the present study were collected on June 2018. The follow–up was completed at the date at which patients died or were censored. The risk of 1–year WRF was assessed using Cox regression analysis expressed as HR, 95% CI, and *P* value. Statistical significance was set at *P* value < 0.05. All statistical analyses were carried out using Stata software version 14 (StataCorp, College Station, TX).

## Data Availability

We agree with the statement.
